# BANΔIT: B’‐Factor Analysis for Drug Design and Structural Biology

**DOI:** 10.1002/minf.202000144

**Published:** 2020-09-06

**Authors:** Fabian Barthels, Tanja Schirmeister, Christian Kersten

**Affiliations:** ^1^ Institute for Pharmaceutical and Biomedical Sciences Johannes Gutenberg-Universität Mainz Staudingerweg 5 55128 Mainz Germany

**Keywords:** B-factor, Protein flexibility, Molecular modeling, Drug design, Bioinformatics

## Abstract

The analysis of B‐factor profiles from X‐ray protein structures can be utilized for structure‐based drug design since protein mobility changes have been associated with the quality of protein‐ligand interactions. With the BANΔIT (B’‐factor analysis and ΔB’ interpretation toolkit), we have developed a JavaScript‐based browser application that provides a graphical user interface for the normalization and analysis of B’‐factor profiles. To emphasize the usability for rational drug design applications, we have analyzed a selection of crystallographic protein‐ligand complexes and have given exemplary conclusions for further drug optimization including the development of a B’‐factor‐supported pharmacophore model for SARS CoV‐2 main protease inhibitors. BANΔIT is available online at https://bandit.uni‐mainz.de. The source code can be downloaded from https://github.com/FBarthels/BANDIT.

## Graphical abstract

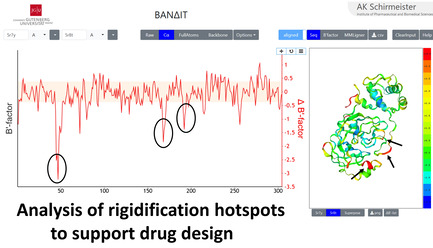

The inherent mobility of proteins is a long‐standing challenge in drug design, but a summary of recent examples showed that the understanding of structural‐dynamic processes can be exploited for rational drug design.^[1]^ Analysis of protein crystal structures is a routine tool for ligand studies. In structure‐based drug design, focus has been put into understanding of the molecular interactions from the 3D‐coordinates, but X‐ray crystal structures naturally also include atomic displacement factors, known as the B‐, Debye‐Waller‐ or temperature factors, which give atomic resolution information on the mobility in the structure.^[2]^ However, the distribution of raw B‐factors is irregular comparing different crystallographic sets, because these are highly influenced by the resolution, crystal packing and the quality of the refinement methods used.^[3]^ Due to these circumstances, it is necessary to normalize B‐factors before they can be compared between different protein structures. The normalized B‐factor (B’‐factor) is a statistic expression of the raw experimental B‐factor for which different calculation methods have been developed.[^4^, ^5^, ^6^]

B’‐factor analysis was previously used to analyze the distribution of active site vs. non‐active site residues,^[7]^ to investigate the flexibility at protein‐DNA interfaces,^[8]^ to differentiate between protein binding sites and crystal‐packing contacts,^[9]^ to estimate protein‐ligand binding affinities,^[10]^ and many other applications summarized in reviews.^[11]^ Recently, this methodology has also been explored for rational drug design because changes in B’‐factors indicate an enhancement or weakening of molecular interactions on an atomic resolution level.[^12^, ^13^, ^14^] The binding of reversible ligands to their targets usually leads to a rigidification of the protein scaffold and manifests itself in a reduction of the B’‐factor which was found to be in approximate correlation to the binding strength of the ligand.^[15]^

The BANΔIT toolkit (https://bandit.uni‐mainz.de) enables facile B’‐factor analysis for users from medicinal and biological chemistry fields by accessing the graphical user interface through a web browser. For offline usage, the underlying source code can be downloaded from https://github.com/FBarthels/BANDIT. The open‐source program package was realized in a client‐side dynamic website environment with HTML/JavaScript and is distributed under LGPL license. Even if the program is accessed via the web appearance, the client‐side content is generated on the user‘s local computer. By this, the submitted crystallographic data never leaves the user‘s computer, an important implication that underlies the fact many solved crystal structures are confidential. Besides data security, this approach is robust, offers excellent cross‐platform usability and the client‐side rendering takes less than a second. The BANΔIT implementation includes the following modules:

**(i) Creating a B‐factor record set from a PDB‐file**: The parse pdb library was developed to create a JavaScript *recordSet*, which is used for handling of either local PDB‐files or fetching from the rcsb.org repository. The respective *fileData* is transferred to a *parseBuffer*, parsed by the library and finally accessible through a *pdbObject*.

Crystallographic B‐factors are a quantity with atomic resolution, i. e. there is an individual B‐factor for each heavy atom of a structure. However, the most common approach to study protein mobility is the residue‐wise analysis of B′‐factors as the flexibility index. The choice of which B‐factors should represent a single residue is guided by the respective application. Thus, the *tempFactors* (B‐factors) specified by the ATOM‐records can be selectively extracted from a *recordSet*. The comparison of C_α_ B’‐factor profiles is the gold standard in the characterization of backbone mobility that results from protein dynamics, but the normalization of an averaged value over all backbone heavy atoms (N, C_α_, C, O) has also been reported in representative studies.^[16]^ For the analysis of side‐chain mobility, all heavy atoms of the structure were considered for B‐factor normalization.[^12^, ^17^]

The choice of the input atoms has further implications: Amino acid side chains might be present in alternate locations. By default, the most frequent location is considered for a residue, but it is also possible to determine an averaged B’‐factor using the occupancy *π* of all alternate locations *l* (eq. 1.

(1) $$B{}^{'}\left(i\right)=\sum \pi \left(l\right)B{}^{'}\left(i,l\right)$$

**(ii) Normalization of B‐factors**: B‐factor normalization is the transformation of experimental B‐factors so that the resulting distribution is defined in terms of the expected value and the variance, which allows the comparison of B’‐factors for different datasets in a way that eliminates gross influences. The normalization procedure is carried out by the process pdb library and the normalized data are transferred to a *tempSet*, i. e. a *recordSet* of B′‐factors per residue.

The first literature‐described algorithm for B‐factor normalization was proposed by Karplus and Schulz and related the experimental B‐factor of a residue *i* to the arithmetic mean of all B‐factors in a structure (eq. 2.^[4]^

(2) $$B{}^{'}\left(i\right)=\frac{B\left(i\right)+D}{\left[\frac{1}{N}\sum_{i=1}^{N}B\left(i\right)\right]+D}$$

The value of *D* for a given structure was iterated in such a way that the root mean square deviation of the resulting B’‐values was equal to 0.3 (eq. 3). This algorithm was previously found to be useful to correlate mobility to different amino acids,[^4^, ^18^] however, in recent times it was largely replaced by other methods.

(3) $$0.3=\sqrt{\frac{1}{N}\sum_{i=1}^{N}{\left(\bar{B{}^{'}}-B{}^{'}\left(i\right)\right)}^{2}}$$

A more recent normalization method for B‐factors is the z‐transformation that relates the arithmetic mean to the standard deviation (eq. 4). B’‐factors normalized with this method show zero mean and unit variance.^[5]^

(4) $$B{}^{'}\left(i\right)=\frac{B\left(i\right)-\frac{1}{N}\sum_{i=1}^{N}B\left(i\right)}{\sqrt{\frac{1}{N-1}\sum_{i=1}^{N}{\left(\bar{B}-B\left(i\right)\right)}^{2}}}$$

However, both estimators used in the standard z‐score, the sample mean and the standard deviation, can be disturbed by even a single outlier value. Smith *et al*. found that experimental B‐factors follow not a normal distribution, but a Gumbel distribution.^[6]^ They developed an approach for the identification of outliers by the median absolute deviation (*MAD*) as a robust measure for the variability of experimental B‐factors around the median $\tilde{B}$ (eq. 5). Following the recommendation of Iglewicz and Hoaglin, a modified z‐score cut‐off value of *M(i)*>3.5 was chosen to label B‐factor outliers (eq. 6.^[19]^

(5) $$MAD=median\left[\sqrt{{\left(B\left(i\right)-\tilde{B}\right)}^{2}}\right]$$

(6) $$M\left(i\right)=\frac{0.674\cdot (B\left(i\right)-\tilde{B})}{MAD}$$

With raw B‐factors filtered for outliers, a standard z‐score with the arithmetic mean ${\bar{B}}_{noout}$ and the standard deviation $\sigma_{noout}$ can be calculated (eq. 7.

(7) $$B{}^{'}\left(i\right)=\frac{B\left(i\right)-{\bar{B}}_{noout}}{\sigma_{noout}}$$

IBM developed a particularly robust modified z‐transformation algorithm (MAD_E_ method) which completely relies on the median $\tilde{B}$ for calculating the z‐score.^[20]^ Depending on the value of the *MAD*, modified z‐scores were calculated in one of two ways (eq. 8). Although this approach has found its way into the B‐factor literature only rarely,^[13]^ it might be advantageous because it is the least influenced by outliers.

(8) $$B{}^{'}\left(i\right)=\left\{\begin{array}{c} \frac{B\left(i\right)-\tilde{B}}{\frac{1.235}{N}\sum_{i=1}^{N}{\left(B\left(i\right)-\tilde{B}\right)}^{2}},\;MAD=0\\ \frac{B\left(i\right)-\tilde{B}}{1.486\cdot MAD}\;\;\;MAD\neq 0 \end{array}\right.$$

**(iii) Post‐processing of B’‐factors**: B’‐factors in a *tempSet* can optionally be post‐processed with various analytical methods. Representative examples have been included: E.g. residue‐wise determination of RMSF‐values from MD simulations are often weighted proportionally to the atomic masses of the included atoms. A similar approach has been proposed for the prediction of B‐factors from RMSF‐values.^[21]^ The atoms *a* of a residue *i* can therefore optionally be weighted to their molecular weight *M(a)* (eq. 9). For ordinary residues the difference might be small (C=12 u–S=32 u), but for selenomethionines (Se=79 u) and high‐resolution X‐ray structures which enable the positioning of hydrogen atoms (H=1 u) significant deviations are known[^22^, ^23^]

(9) $$B{}^{'}\left(i,a\right)=\frac{1}{M\left(i\right)}\sum M\left(a\right)B{}^{'}\left(i,a\right)$$

If a B’‐factor profile resolution greater than a single residue is desired, the fluctuating atomic resolution of B′‐factors was found to be a hindrance, e. g. if B’‐factors are used for the characterization of secondary structure motives. Since abrupt changes in flexibility within a closed backbone sequence are physically not to be expected, a smoothing method for B′‐factors was implemented.^[24]^ Smoothed $B_{sm}^{'}$ ‐factors can be calculated by a moving average with a variable residue window size *n* (eq. 10.

(10) $$B_{sm}^{{}^{'}}\left(i\right)=\frac{1}{n}\sum_{k=0}^{n-1}B{}^{'}\left(i-k\right)$$

**(iv) Alignment of structures**: B‐factors are primarily normalized to be compared between different data sets. It is possible to calculate the difference ${\Delta B{}^{'}=B}_{complex}^{'}-B_{apo}^{'}$ only if two corresponding B′‐factors exist, thus, incompletely resolved atomic records must be excluded by the process pdb library. To derive structural‐biologically relevant statements ΔB′‐values have to be finally checked for their significance in the ΔB′‐population (p<0.05).[^12^, ^25^]

To perform a comparative analysis, an alignment between the target data sets may be necessary. Based on the optimization algorithm proposed by Needleman and Wunsch a common sequence‐based alignment was implemented for standard use on nearly identical proteins.^[27]^ Furthermore, based on the MMLigner package,^[28]^ which allows structural alignments built on Bayesian and information‐theoretic principles, the possibility was implemented to align structurally conserved but sequentially different proteins. Since the MMLigner program has been developed in C++, the complementary JavaScript code was ported with the LLMV‐JS compiler Emscriptem.^[29]^

However, the MMLigner algorithm is computationally quite demanding, hence, an accelerated B′‐factor‐based dynamic programming procedure for a three‐dimensional fuzzy alignment was developed. A method of Blankenbecler *et al*. was adapted that matched residues by comparing structural categories by an optimization algorithm (eq. 11.^[30]^

(11) $$F\left(i,j\right)=max\left\{\begin{array}{c} F\left(i-1,j-1\right)+w\left(x_{i},y_{i}\right)\\ F\left(i-1,j\right)+f\\ F\left(i,j-1\right)+f \end{array}\right.$$

Based on the B′‐factors, a scoring function $w(x_{i},y_{i})$ was developed which assigns each residue to a category (A–G) according to its absolute B′‐factor value (F>2.8>D>1.4>B>0.6>A>−0.6>C>−1.4>E>−2.8>G). Scores were calculated based on the similarity of the B′‐factor categories (e. g. $w\left(A,A\right)=3,\;w\left(A,B\right)=2,\;w\left(A,E\right)=1,\;w\left(A,F\right)=0).$ Gaps were penalized relatively high (f=3) to ensure correct alignment in domains with varying mobility.

**(v) Visualization of the results**: The graphical presentation of the calculated B’‐factors was realized in the HTML and JavaScript environment for display in a web browser. The free‐for‐academic charting API canvasJS (https://canvasjs.com/) was used for data presentation and interactive B‐factor profile analysis. The NGL WebGL‐based molecular viewer was implemented for the interactive display of protein structures.^[26]^ Furthermore, the data can also be exported in pdb‐ or csv‐format for external processing. A screen dump of the toolkit's user interface and usage instructions are shown in Figure 1. For interaction hotspot analysis of B’‐factor profiles across multiple structures these can be visualized in a heatmap format with the open‐source charting API ApexCharts (https://apexcharts.com/).

**Figure 1:**
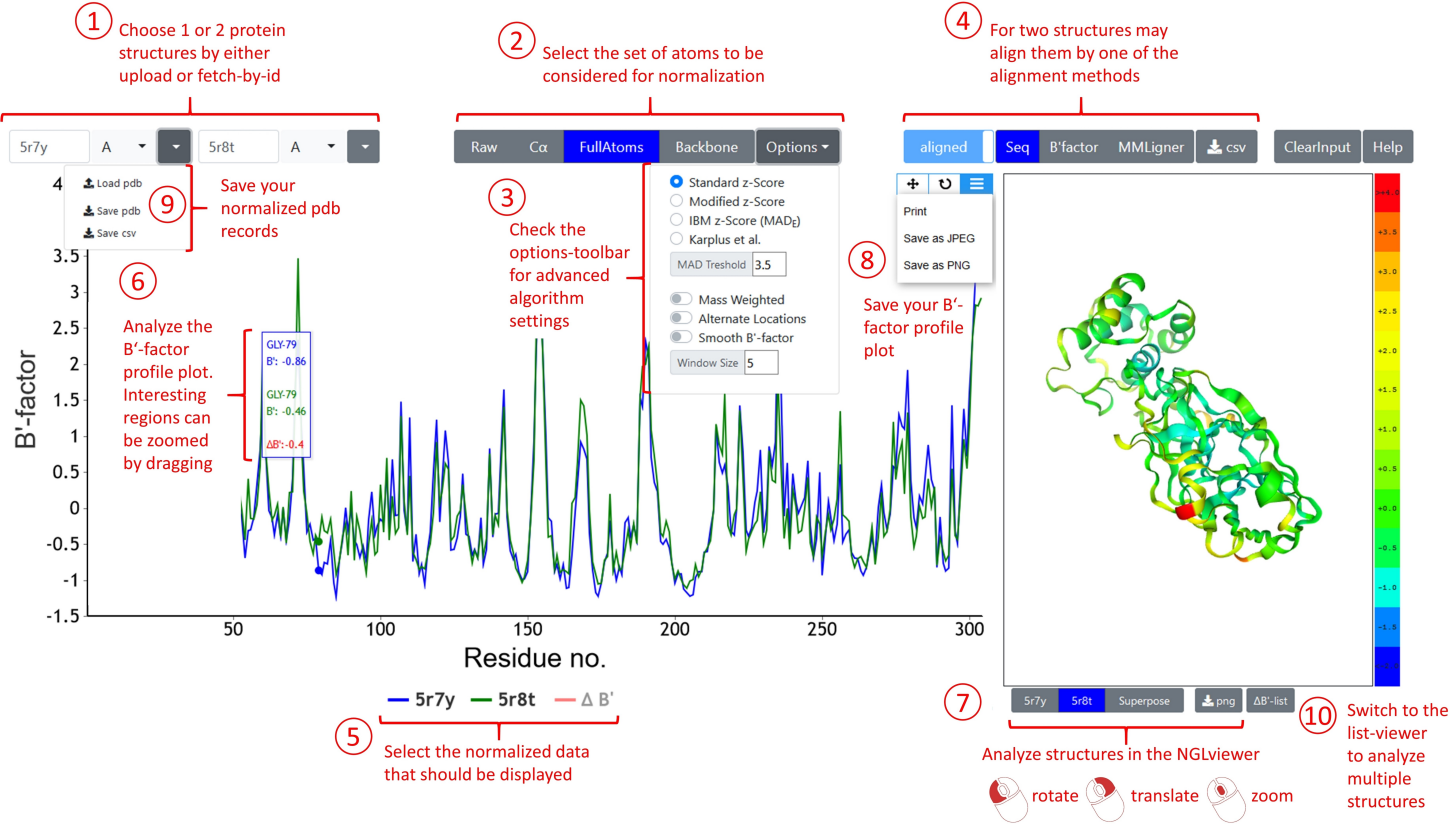
User interface of the BANΔIT with numbered instruction steps for pairwise analysis of B’‐factor profiles. (1) Choose 1 or 2 protein structures by either upload or fetch‐by‐id. (2) Select the set of atoms to be considered for normalization. (3) Check the options‐toolbar for advanced algorithm settings. (4) For two structures may align them by one of the alignment methods. (5) Select the normalized data that should be displayed. (6) Analyze the B‘‐factor profile plot. Interesting regions can be zoomed by dragging. (7) Analyze the 3D‐models colored by their B’‐factors in the NGLviewer.^[26]^ (8) Save the B‘‐factor profile plot. (9) Save the normalized PDB‐records. (10) Switch to the list‐viewer interface for multiple structure alignment and heatmap visualization

To demonstrate the scope of BANΔIT, we have briefly analyzed a selection of literature examples that have investigated the dynamic processes of protein‐ligand binding by conventional techniques like nuclear magnet resonance spectroscopy (NMR) and molecular dynamic (MD) simulations. With our toolkit, we aimed to reproduce the statements regarding dynamics and flexibility by a retrospective analysis of corresponding crystallographic B‐factors which have not yet received any attention. To demonstrate the applicability for relevant prospective studies, we have also developed a B′‐factor‐supported pharmacophore model for SARS CoV‐2 main protease inhibitors based on a recently solved crystallographic fragment screening dataset.

**Example 1 Tyrosine phosphatase 1E PDZ domain**: Dhulesia *et al*. described the changes in dynamic processes that occur in the second PDZ domain of the human tyrosine phosphatase 1E (PTP1E) upon binding of the small peptide RA‐GEF2 by protein NMR and MD simulations.^[31]^ The selective inhibition of PDZ‐mediated protein‐protein interactions was considered to be an approach for drug development in cancers that are based on abnormal activities in the underlying pathways.^[32]^ The rational design of inhibitors of protein‐protein interactions is still considered difficult because of the inherent protein flexibility.[^33^, ^34^] We see a special potential in our toolkit supporting rational drug design in this context.

The B’‐factor analysis of the PTP1E crystal structures in the absence or presence of the RA‐GEF2 ligand perfectly reflects the same results of the NMR‐order parameter restrained MD calculations.^[35]^ The β2/β3‐loop (T28–G34) and a distal surface region (L66–E76) are significantly rigidified once the RA‐GEF2 ligand binds (Figure 2A).

**Figure 2:**
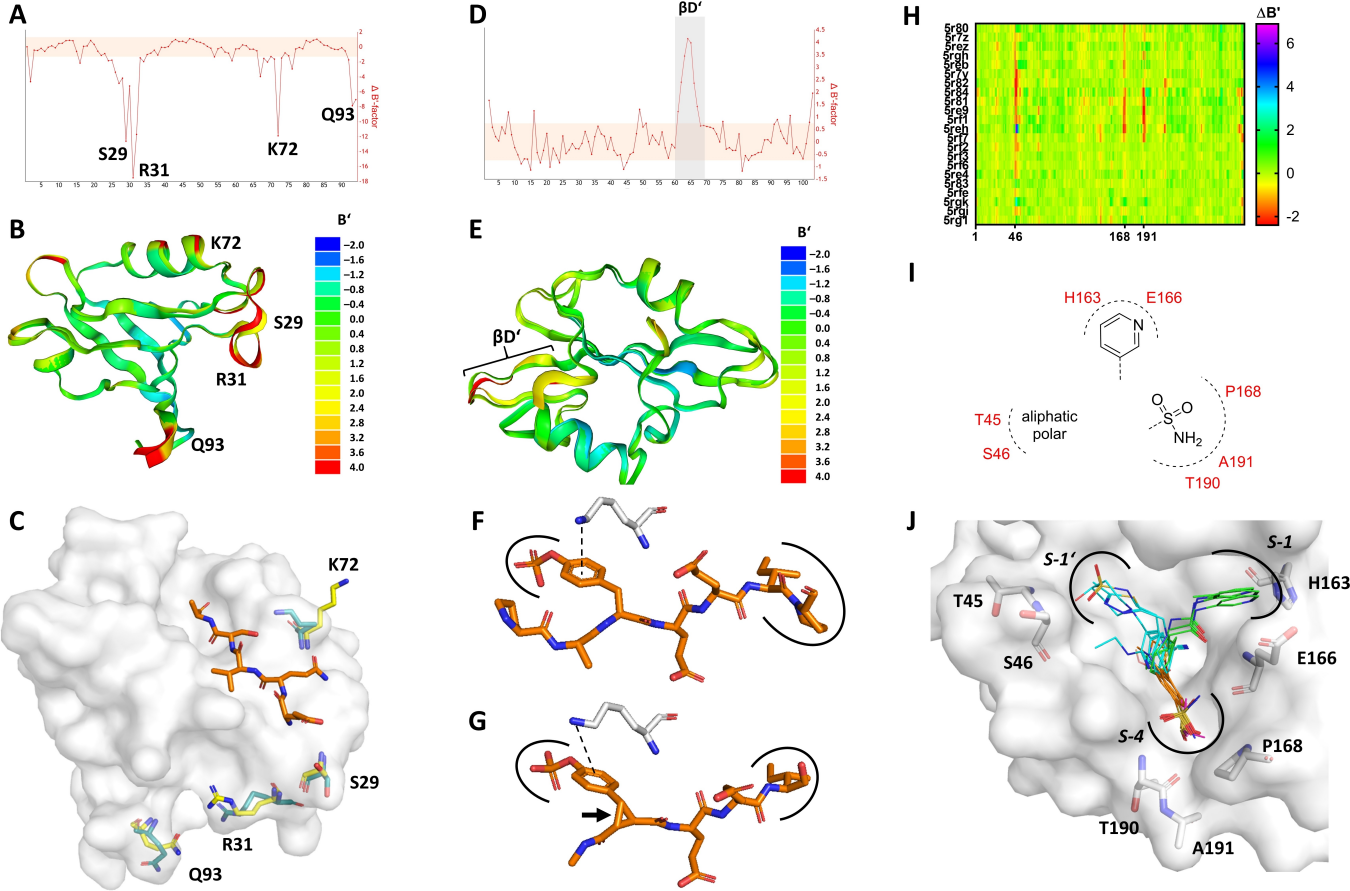
Presentation of the B′‐factor analysis from representative drug design examples. (**A**) Plot of ΔB′ for PTP1E apo‐structure (PDB: 3LNX) *vs*. PTP1E in complex with the RA‐GEF ligand (PDB: 3LNY). ΔB′‐values outside the salmon‐colored horizontal box are statistically significant (p<0.05). (**B**) Superposed crystal structures for PTP1E with and without the RA‐GEF ligand colored by the B’‐factors. (**C**) Representation of the most rigidified residues in the RA‐GEF2 PTP1E complex. *Turquoise*: PTP1E apo‐structure; *Yellow*: PTP1E holo‐structure; O*range*: RA‐GEF2 peptide ligand. (**D**) Plot of ΔB′ for the Src kinase in complex with the conformationally constrained ligand (PDB: 1IS0) *vs*. Src in complex with the natural ligand (PDB: 1SPS). (**E**) Superposed crystal structures for the respective Src complexes with both ligands colored by the B’‐factors. (**F**) The natural phosphopeptide ligand reveals a favourable cation‐π interaction with K60. (**G**) The cyclopropyl constraint (*arrow*) leads to a suboptimal cation‐π interaction, which induces increased mobility in the neighboring residues (K60–L64). (**H**) B’‐factor analysis of SARS CoV‐2 main protease in complex with multiple fragments. Clustering of 22 non‐covalent complexes *vs*. the non‐liganded apo‐structure (PDB: 5R8T). (**I**) Development of a B’‐factor‐based pharmacophore hypothesis. (**J**) Superposition of selected crystal structures from a crystallographic fragment screening to the SARS CoV‐2 main protease. In the center, aromatic structural elements are predominant. Sulfonamides interact with T190, A191 and P168 (PDB: 5R80, 5R81, 5RF1; Carbons colored in orange). DMSO molecules were also found in this S‐4 pocket (PDB: 5REH, 5R82, 5RE9; Carbons colored in magenta). Various polar fragments interact with T45, S46 (PDB: 5REB, 5R7Y, 5R82, 5RGH; Carbons colored in cyan). Pyridine containing ligands show interactions with H163 and E166 (PDB: 5RE4, 5R83, 5R84; Carbons colored in green).

In the ribbon model with a coloring relative to the C_α_ B’‐factor of a residue (B‐factor putty) the key mediators of rigidification (S29, R31, K72 and Q93) can be visualized (Figure 2B). This information might be useful to determine which residues should be kept flexible in an induced‐fit docking protocol.^[36]^ In cases where residues are characterized by high B’‐factors, several conformations will probably exist in solution. A mechanistic model for ligand binding was developed from the rigidified residues: K72, which protrudes into the binding pocket in the apo‐structure, is displaced by the ligand and rigidified via backbone interactions. S29 interacts with the ligand via the side‐chain hydroxyl group and thus stabilizes the entire β2/β3‐loop, resulting in a reorientation of R31 ultimately interacting with the distal Q93 (Figure 2C).

**Example 2 Src kinase SH2 domain**: In extensive NMR and MD studies of Src kinase ligand complexes, it was found that a comparison of natural and conformationally constrained ligands leads to NMR chemical shift deviations across the binding site. MD simulations supported these findings and the investigators concluded that the observed enthalpic penalty is a result of increased flexibility in the binding site.^[37]^ By B′‐factor analysis we could confirm these results and showed that there is a significant dynamic increase in the βD′‐sheet (K60–L64, Figure 2D&E). Inspection of the ligand poses revealed this is a consequence of the poor cation‐π interaction of K60 with the constrained ligand (Figure 2F&G). By this example, we showed B′‐factor analysis can be helpful to understand thermodynamic ligand binding issues. Based on the results, it might be advisable to place the conformational constrain at a different position in the ligand.

**Example 3 SARS CoV‐2 main protease**: At the Diamond Light Source of the UK national synchrotron facility a high throughput crystallographic fragment screening was performed (yet unpublished^[38]^), which solved 44 covalent and 22 non‐covalent fragment complexes of the SARS CoV‐2 main protease (Mpro). Since a drug to treat COVID‐19 is desperately needed in 2020,^[39]^ our toolkit was used to develop a B′‐factor‐supported pharmacophore model of the 22 non‐covalent fragment complexes in the active site binding pocket.

While it can be easily seen from the crystal structures that in the center of the active site pocket (S2‐ and S3‐site) an overlap of aromatic core structures dominates, superposition of all fragment poses leads to a diverse pattern of interactions with distal residues of the substrate‐binding site.^[38]^ Common practice would be to examine the set of fragments for a superposition of structural features,^[40]^ but this does not allow quantification of the interaction quality. Therefore, we have chosen a different approach and calculated ΔB′‐factor profiles of all non‐covalent fragment complexes versus the apo‐structure. Hotspots of mobility changes due to specific ligand features were clustered and presented in a heat map (Figure 2H).

In total, three hotspots for ligand‐induced protein rigidification were identified. We found that sulfonamides strongly stabilize the backbone atoms of T190, A191 and the side chain atoms of P168 in the S4‐pocket. For some crystal structures where this pocket is not occupied by a ligand, analogously a DMSO molecule is located in this pocket, which has also a rigidifying effect. In general, the binding of sulfoxides and sulfonamides to this pocket seems to be preferred. Secondly, T45 and S46 at the edge of the S1’‐pocket are also strongly rigidified by ligands that encompass various polar‐decorated structural elements. However, a clear structural trend was not identified. Primarily aliphatic scaffolds with polar decoration such as 4‐hydroxypiperidines, N‐ethylmethanesulfonamides, 2‐ethylamino pyridines or 2‐methylthiadiazoles should be considered. A single complex (PDB: 5REH) showed significantly increased mobility of S46. However, since this ligand has no contact with S46 and a large part of the ligand is solvent‐exposed, we believe that this is an artifact without relevance for ligand binding.

In contrast, the S1‐pocket (H163, E166) was often occupied by heteroaromatic structural features such as pyridines. However, no significant change in the ΔB′ of the surrounding residues was observed. This can be explained by the fact that a water network is perturbated when ligands bind into this pocket.^[41]^ A prospective analysis of this putative entropically dominated binding by water displacement is outside the scope of B′‐factor analysis. From the combined results a B′‐factor‐supported pharmacophore model was developed, which might be complementary to upcoming conventional fragment clustering, linking and merging efforts (Figure 2I&J).^[42]^

In conclusion, we demonstrated the BANΔIT can be used for rational drug design applications. It provides an additional tool based on a measure that is already existing in crystal structures. Especially in the combination with NMR‐ and MD‐experiments, pioneering results in drug design can be expected in the future.

## Conflict of Interest

None declared.
